# Effects of Subchronic Treatment with Ibuprofen and Nimesulide on Spatial Memory and NMDAR Subunits Expression in Aged Rats 

**Published:** 2013

**Authors:** Ozlem Ozturk Bilgin, Duygu Kumbul Doguc, Irfan Altuntas, Recep Sutcu, Namık Delibas

**Affiliations:** a *Konak Hospital, Medical Biochemistry Department, Izmit, Turkey. *; b *Suleyman Demirel University, Medical Faculty, Medical Biochemistry Department, Isparta, Turkey. *; c *Mugla University, Medical Faculty, Medical Biochemistry Department, Mugla, Turkey. *; d *Katip Celebi University, Medical Faculty, Medical Biochemistry Department, İzmir, Turkey.*; e *Hacettepe University, Medical Faculty, Medical Biochemistry Department, Ankara, Turkey. *

**Keywords:** Nonsteroidal antiinflammatory drug (NSAID), Cyclooxygenase (COX), NR2A and NR2B, Spatial memory, Radial arm maze (RAM)

## Abstract

Several studies point to an important function of cyclooxygenase (COX) and prostaglandin signaling in models of synaptic plasticity which is associated with *N*-methyl-D-aspartate receptors (NMDARs). Cyclooxygenase gene is suggested to be an immediate early gene that is tightly regulated in neurons by NMDA dependent synaptic activity. Nonsteroid Antiinflammatory Drugs (NSAIDs) exert their antiinflammatory effect by the inhibion of COX have controversial effects on learning and memory. We administered ibuprofen as a non-selective COX-2 inhibitor and nimesulide as a selective COX-2 inhibitor for 8 weeks for determining the cognitive impact of subchronic administration of NSAIDs to aged rats. Wistar albino rats (16 mo, n = 30) were separated into control (n = 10), ibuprofen (n = 10) and nimesulide (n = 10) treated groups. First we evaluated hippocampus-dependent spatial memory in the radial arm maze (RAM) and than we evaluated the expression of the NMDAR subunits, NR2A and NR2B by western blotting to see if their expressions are effected by subchronic administration with these drugs. Ibuprofen and nimesulide treated rats completed the task in a statistically significant shorter time when compared with control group (p < 0.01), but there was no statistically significant difference between groups about choice accuracy data in RAM. Furthermore, no statistically significant difference was detected for the protein expressions of NR2A and NR2B of the subjects. Oral administration of ibuprofen and nimesulide for 8 weeks showed no impairment but partly improved spatial memory.

## Introduction

The hippocampus, a part of the limbic system, is found to be necessary for several types of learning and memory formation in rats and other mammals (1, 2). The *N*-methyl-D-aspartate receptor (NMDAR) which is highly expressed in the hippocampus is implicated in some types of long term potentiation (LTP) that might underlie spatial learning and memory (3). 

Cyclooxygenase (COX) is the key enzyme that converts arachidonic acid (AA) to prostaglandins (PGs) and the target enzyme of nonsteroidal antiinflammatory drugs (NSAID) (4). Cyclooxygenase isoform, cyclooxygenase-1 (COX-1) is the constitutive form of cyclooxygenase and performs a housekeeping function to synthesize PGs, involved in regulating normal cellular activities (4-8). In contrast, the COX-2 promoter is not basally active in most cell types, but can be strongly and rapidly induced by growth factors and proinflammatory mediators (9, 10). The brain expresses both the isoforms. COX-2 is expressed in discrete populations of neurons and is enriched in the cortex and hippocampus (11) and has been implicated in brain functions and in neurologic disorders, including stroke, seizures, and Alzheimer’s Disease (AD) (5, 12). Within the hippocampus, COX-2 protein is observed especially where the glutamatergic neurons are selectively colocalized (6) and neuronal COX-2 expression appears to be coupled to excitatory neuronal activity which is suggested to be dependent on NMDAR activity (13, 14). Previous research has employed different kinds of NSAIDs (piroxicam, NS-398, celecoxib, indomethasin) to examine the effect on spatial memory in rats. The results support the idea that COX-2 is probably involved in the physiological mechanisms underlying memory formation (4, 8, 11). Although COX-2 appears to facilitate cognition under normal conditions, studies in the aged rats suggests that dysregulation or overexpression of COX-2 function is detrimental to memory consolidation (15-17). Recent studies have also indicated that in addition to the overexpression of COX-2 mRNA and protein (18), COX-1 has a previously unrecognized proinflammatory role in the pathophysiology of acute and chronic neurological disorders such as AD, Parkinson Disease, HIV-associated dementia (19-21). A selective increase in COX-1 mRNA expression in the hippocampus of aged rats was shown which was possibly causing an increased susceptibility to neuroinflammation (22) and also COX-1-expressing microglia are found surrounding the amyloid plaques in the AD brain indicating a role of this isoform in the pathophysiology of the disease (21, 23).

Based on the compelling evidence that inflammatory processes are involved in the pathogenesis of AD, it has been hypothesized that NSAIDs might slow the onset and progression of AD (24, 25). For this purpose ibuprofen has been used in many clinical and experimental investigations and some benefial effects on AD related pathological constituents such as reduction of the *β *amiloid plaques and amyloid precursor protein (26-32) and blockade of Rho protein (29, 33) has been demostrated. Studies with nimesulide in rodents indicate that the inhibition of COX-2 activity attenuates brain inflammation associated with excitotoxic damage (34, 35). Aisen *et al*. reported that nimesulide has no short-term cognitive or behavioral toxicity, nor any clear symptomatic benefit on the manifestations of AD (36). However, the subchronic effects of ibuprofen and also nimesulide on spatial memory in aged brain and their probable effects on NMDARs hasn’t been investigated. 

In this study we investigated the effects of subchronic administration of ibuprofen as a non-selective COX inhibitor and nimesulide as a selective COX-2 inhibitor, on spatial memory in the eight arm radial maze. In addition, the effect of drug treatment on protein expression of NR2A and NR2B, the subunits of NMDARs, was examined.

## Experimental

*Animals*

Wistar albino male rats (16 months (mo), n = 30) were separated into control (n = 10), ibuprofen (n = 10) and nimesulide (n = 10) treated groups. The animals were purchased from Animal Investigation Laboratory of Suleyman Demirel University and they were housed in groups of five. The rats were given *ad libitum *access to water; however, their weights were maintained at 80 % of their free-feeding levels. They were fed daily after testing. Because rats are nocturnal animals, we reversed their day-light cycle and testing occurred during the dark phase which is their most active period (37). Testing also occurred under dim-light conditions.

The animals were handled under the prescriptions for animal care and experimentation of the pertinent European Communities Council Directive (86/609/EEC), and all the procedures were approved by the Ethical Committee of the Suleyman Demirel University. *Radial arm maze training*

The maze was made of black coloured plexiglass and consisted of a center platform 35 cm in diameter, and eight extending arms (10*80 cm). The maze was elevated 80 cm from the floor and was located in a room with many extramaze visual cues. Food cups, located at the ends of each of the arms, were baited with a piece of corn flakes. All arms were baited prior to testing and no arm was rebaited after testing began. The maze was wiped off with a towel between each training session. Prior to testing the rat was placed in an opaque cylinder, approximately 30 cm in diameter that was placed in the central area of the maze for 5 sec. Timing began after the cylinder was lifted and the rat was free to explore. Arm choices were recorded after all four rat paws crossed completely into the arm. The rat had a maximum of 300 sec. to find all the reinforcers. If the rat reentered an arm, it was counted as an error. This procedure tested working memory, that is, memory for cues encountered during a specific trial of a task. The measure of choice accuracy or entries to repeat (ETR) was the number of correct entries made before an error was made. The measure of response latency was seconds per entry, the total length of the session, divided by the number of entries made. The rats were tested 5 days/week (36). After 20 sessions of acquisition training on the radial-arm maze, rats consistently scored high ETRs (6–9) and drug administration started and continued for 8 weeks. At the end of the drug administration period, animals were again tested on the RAM and the data for this probed trial was used for statistical comparisons. Animals were16 months old at the beginning and 18 months when tested. The pool was surrounded by four halogen lights which were directed to the walls that surrounded the room in order to prevent direct lighting to the rats and to provide moderate dim light during testing. We recorded the training and testing periods with a ceiling-mounted videocamera (Sony SSC-DC398P, Japan) and used an automated computer-based system (SMART Version 2.0) to quantify the trace of rats in the eight-arm radial maze during the test. This system allowed us to monitor each rat in the maze with a camera equipped with a personal computer. 

*Drugs*

Ibuprofen (sodium salt), nimesulide, ketamine, and xylazine were purchased from Sigma Chemical Company (St Louis, MO, USA). Ibuprofen group received 40 mg/kg/day ibuprofen (38), nimesulide group received 9 mg/kg/day nimesulide (39) and control group received the same volume of physiological saline over 8 weeks period. Ibuprofen and nimesulide were prepared daily and dissolved in 2 ml distilled water before administration. Ibuprofen and nimesulide were administered by oral gavage to the rats (40). At the end of 8 weeks each rat was anesthetized separately by injecting intraperitoneal 2% xylazine (10 mg/kg) and then 10% ketamin (80 mg/kg). This anesthesia gave us 1 h time window to sacrifice the animals.

*Tissue collection*

Rats were decapitated and the brain was rapidly removed. The hippocampus were dissected on an apparatus which was icy and wetted with phosphate buffer (50 mM) and frozen in the eppendorfs which were filled with phosphate buffer (50 mM). Samples were stored at −80°C until assayed (41).

*Western blotting*

**Figure 1:**
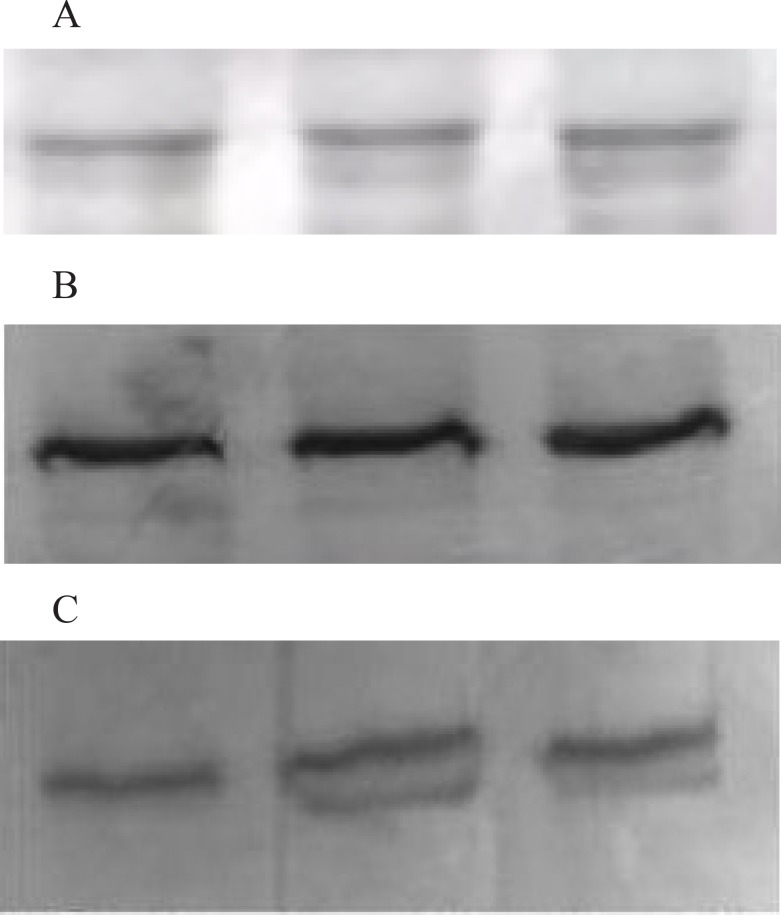
Representative western blotting bands of all groups from hippocampi. Blotting of β-actin was used as an internal standard to confirm equal protein loading and sample transferring. Expression of NR2A and NR2B protein was normalized against that of β-actin. 1A:Western Blotting sample of β-actin First band: Control group; Second band: Nimesulide group; Third band; Ibuprofen group. 1B: Western Blotting sample of NR2A First band: Control group; Second band: Nimesulide group; Third band; Ibuprofen group. 1C: Western Blotting sample of NR2B First band: Control group; Second band: Nimesulide group, Third band; Ibuprofen group. Representative western blotting bands of all groups from hippocampi. Blotting of β-actin was used as an internal standard to confirm equal protein loading and sample transferring. Expression of NR2A and NR2B protein was normalized against that of β-actin

First protein concentration of in hippocampi were measured (42). Antibodies against NR2A and NR2B were purchased from Sigma Chemical Company (St Louis, MO, USA) and mouse monoclonal antibody to *β*-actin were purchased from Abcam (Cambridge, USA). All other reagents were of analytical grade or the highest grade available. The hippocampi (2-3 animals/preparations) were homogenized in ice-cold buffer [50 mm Tris-HCl (pH 7.5), 0.15 M NaCl, 1% Triton X-100, 1 mM EDTA, 1 mM EGTA, 25 μg/mL leupeptin, 25 μg/mL aprotinin, 1 mM sodium orthovanadate, 10 μM benzamidine and 4 mM p-nitrophenyl phosphate] and an aliquot was taken for protein determination. Equal amounts of protein for each sample (50 μg of protein per lane) were separated by SDS/PAGE on 7.5% minigels, blotted electrophoretically to immobilen membrane, and incubated in tris-buffered saline with Tween 20 (TBST) [50 mm Tris-HCl (pH 7.5-8.0), 150 mM NaCl, and 0.1% Tween 20] containing 3% bovine serum albumin (BSA) for 30 min. Blots were incubated overnight with anti-NR2A (1:3000), anti-NR2B (1:5000) and anti-*β*-actin (1:5000) in 1% BSA. Immunoblotting for *β*-actin was used as an internal standard to confirm equal protein loading and sample transfer (Figure 1A). Blots were subjected to three additional 10-min washings in TBST and were incubated with alkaline phosphatase conjugated monoclonal anti-rabbit IgG (1:10000) in 1% BSA for 1 h at room temperature and 3 additional washings were performed with TBST for 10 min. The membrane was incubated in 20 mL of fresh reagent solution (BCIP/NBT) until color development. Images of immunoblots were analyzed with a computerized image analysis system (Kodak MM 2000 Image Station, USA). SDS-PAGE and Western blot analyses were done on 3 independent hippocampus preparations (2-3 animals/group) (41). 

*Statistical analysis*

The choice accuracy and response latency data were first assessed by Levene homogeneity test and the data were not homogeneous so a non parametric Kruskall Wallis test were used to assess these two data. A p-value of less than 0.05 was considered to be statistically significant. For the significant differences we used Bonferroni corrected Mann-Whitney U tests to determine which group was responsible for the difference. The results were given as mean ± SEM (standart error of mean). A p-value of less than 0.01 was considered to be statistically significant.

NR2A and NR2B protein levels were also assessed by Levene homogeneity test and the data were not homogeneous so Kruskall Wallis Test was used to assess the protein levels. A p-value of less than 0.05 was considered to be statistically significant.

## Results

*Effect of subchronic administration of Ibuprofen and Nimesulide on NR2A and NR2B protein expressions*

**Figure 2:**
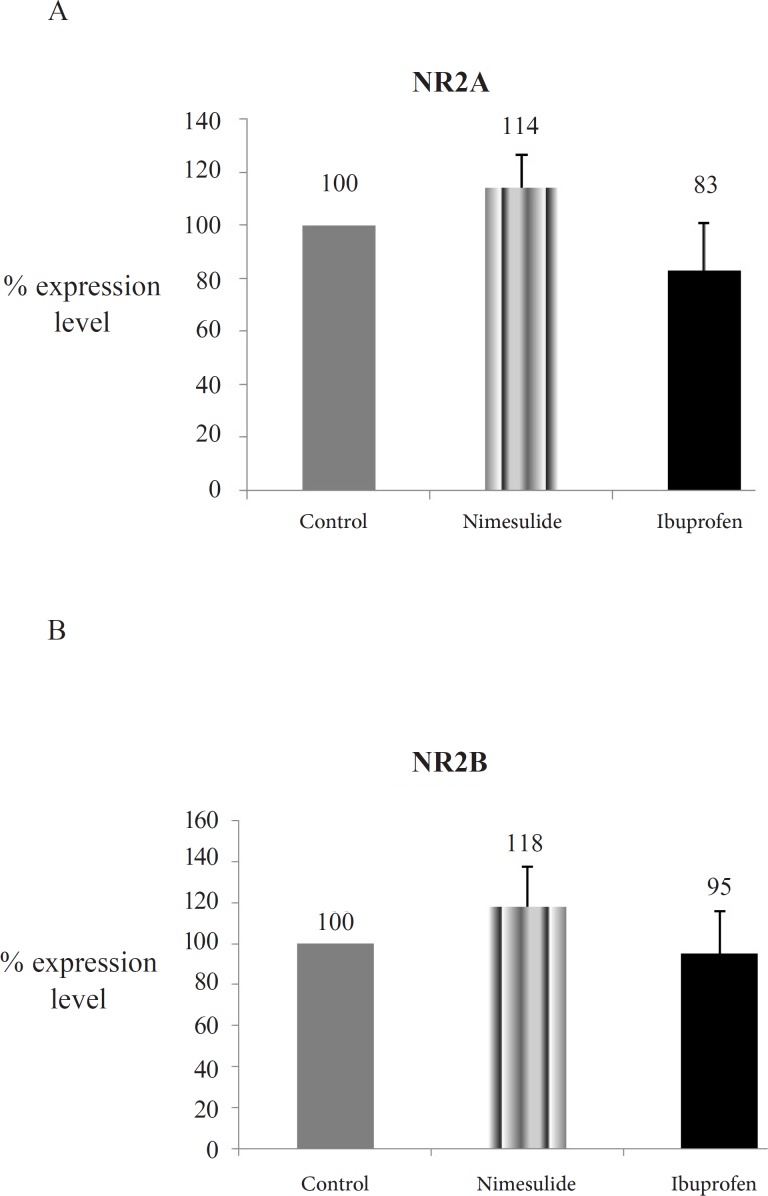
Optic densities of NMDAR subunits protein expressions of the groups. 2A: NR2A protein expressions; 2B: NR2B protein expressions; Explanation of Figure 2A, 2B: NR2A and NR2B levels from hippocampi homogenates were assayed with western blotting. Mean of control group data was assumed as 100 and % concentration values of other groups were given (Data are presented as mean ± SEM).

The NR2A and NR2B protein concentrations of nimesulide and ibuprofen groups showed no difference when compared with control group (p > 0.05) (Figure 1B, Figure 1C, Figure 2A and 2B).

*Effect of Subchronic administration of Ibuprofen and Nimesulide on spatial memory performance (8 arm radial maze)*

**Figure 3:**
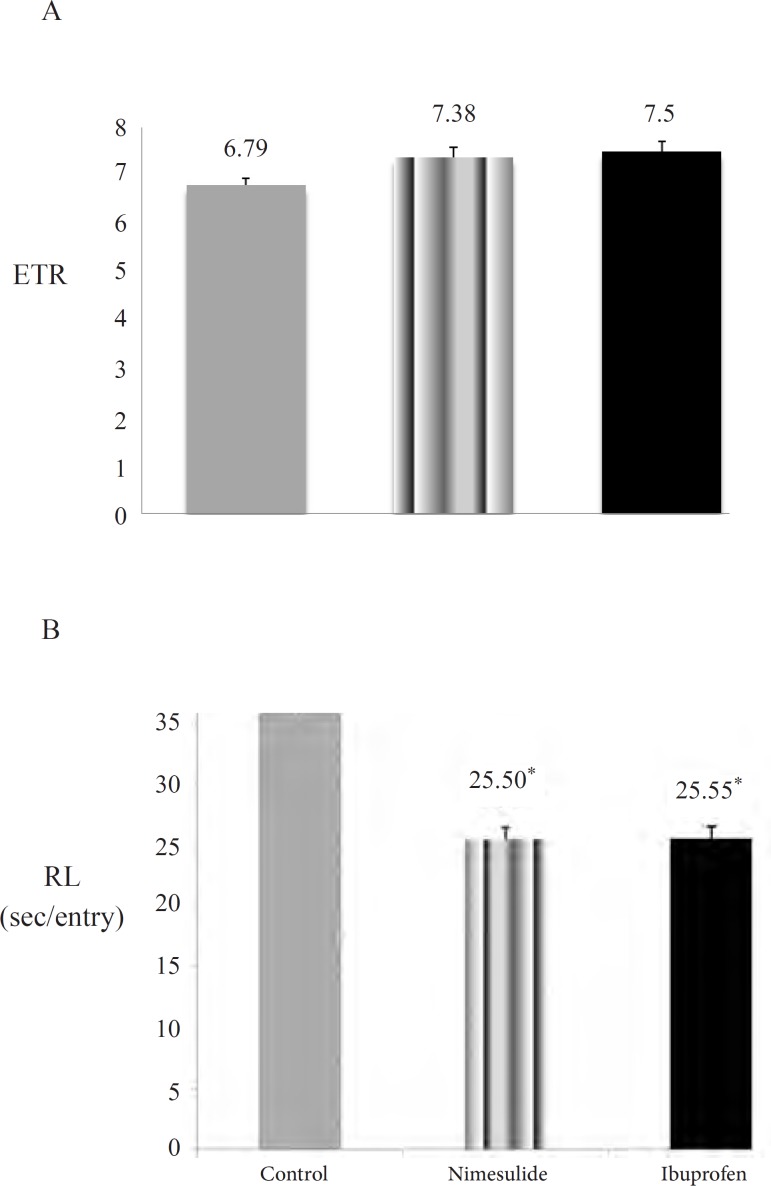
Effects of subchronic nimesulide and ibuprofen administration on spatial memory performance. 3A: choice accuracy (Entries to repeat; ETR) (Data are presented as mean ± SEM). 3B: response latency (RL) (Data are presented as mean ± SEM). ‘*’ expresses the statistically significant value (p <0.01).

While choice accuracy were increased in ibuprofen and nimesulide groups when compared with control group, these differences were not statistically significant (p > 0.01) (Figure 3A). The only significant effect of ibuprofen and nimesulide was on response latencies which was significantly decreased in these two groups when compared with control group (p < 0.01) (Figure 3B).

## Discussion

A considerable body of evidence indicates that COX-2 is involved in memory acquisition (4) and memory consolidation (11, 43). In spite of several studies about this issue, the role of COX, especially COX-2 in learning and memory process is still unclear. Previous works have provided evidence that inhibition of COX can impair memory (2, 4, 8, 11, 44-47) but on the other hand, inhibition of COX is suggested to improve memory (16, 48, 49). Andreasson et al suggested that the effects of COX-2 activity interacted negatively with aging (15). Additionally, Matousek *et al. *showed that COX-1 deletion is sufficient to significantly improve contextual fear learning and abrogate the effect of IL-1 over-expression (50). Some of the investigators have shown NSAID-induced reversal of age-related memory impairments for different behavioral tasks (16, 48, 49). In addition, Casolini *et al*. showed that during aging, the hippocampal content of inflammatory markers such as IL1*β*, TNF*α *were significantly increased. These investigators suggested that dietary administration of NSAID to aged rats attenuated age-related deficits in memory, at least in part, by decreasing inflamatory processes (16, 49). 

Our findings are not strictly in accordance with any of these suggestions. In some of the investigations, administration of different kinds of NSAIDs had an impairment effect on spatial learning in rats, our results do not support the idea that COX impairs memory. This contraversy may be due to the treatment methods and schedule or the age of the animals employed in our study, since aged brain may have different neurochemical features such as increased proinflammatory enzymes, cytokines, and impaired oxidation-antioxidation balance (51). In our study we assessed the spatial memory, by choice accuracy and response latency data on RAM. While choice accuracy data of ibuprofen and nimesulide groups were increased they were not statistically significant when compared with the control group after the subchronic NSAID administration. However, response latencies were significantly improved which indicates that it took shorter time for ibuprofen and nimesulide administered rats to complete the task when compared with the control group. Although it is just a speculative suggestion, prolonged administration period of these drugs might have improved the choice accuracy with response latency together. On the other hand, improvement in the responce latency data might have depended upon the antiinflamatory and analgesic effect of the drugs in aged body joints so their motility performance might have increased. As this is the limitation of our study, the locomotor activity should be evaluated by an additional behavioral test so that the effect of drugs on motility performance should be eliminated. Finally, improvement at response latency has to be supported with choice accuracy data to make a suggestion that subchronic treatment regimen with ibuprofen or with nimesulide in the aged rats may improve spatial learning and memory. These findings are partly in accordance with the investigations that showed the improvements in spatial learning in aged rats. This partial difference may have resulted from the agent that we used or period of administration should be another fact. Since it has been reported that NSAIDs may have additional targets, including NFκB, *γ*-secretase activity and adenosine and cannabinoid receptors (43, 52), we can suggest that different types of NSAIDs may have different kinds of targets in brain and so, may use different mechanisms of action in brain, therefore the effects of these agents on cognitive performance might be different. 

As we mentioned before, the level of COX-2 expression in the hippocampus is correlated with neuronal activity and up-regulation of COX-2 in hippocampal neurons is thought to be dependent on NMDAR activity (14, 17). In recent studies COX-1 is suggested to be involved in NMDA mediated PG production and excitotoxicity as well as LPS-induced neuroinflammation and behavioral changes (20, 50, 53, 54). In addition, in aged rats, state of chronic inflammation is an ongoing process (55) and COX-2 expression is found to be increased with aging (51, 56). The molecular mechanisms underlying this COX-1 and/or COX-2 overexpression remain unclear and we wondered if NMDAR activity would be responsible for this overexpression of COX in aged brain or if subchronic inhibition of COX enzyme, may cause compensational upregulation on NR2A and NR2B protein expressions. For this reason, in this study we have also determined NR2A and NR2B protein levels. However, we have found that NR2A and NR2B receptor expressions were not statistically affected by 8 weeks usage of nonselective COX and selective COX-2 inhibition. There are very few studies which investigated the probable relation between NSAIDs’ effects and NMDARs. Mesches *et al*. administered sulindac as a nonselective NSAID to the aged rats for 2 months and determined the protein concentration of NR1, NR2A and NR2B of the rats (49). Their findings indicated that the administration of sulindac ameliorated age-related decreases in both the NR1 and NR2B subunits but not in NR2A subunit. In our study NR2A and NR2B protein levels were not statistically significant when compared with control group. Although the administration route and duration were similar to Mesches *et al*. (49), we suggest that the contradiction might arise from the difference of NSAID used in this study and the age of rats which can lead to different findings. 

Our results suggest that subchronic usage of ibuprofen and nimesulide in aged brain have shown partly improvement on spatial memory. However, our findings are insuffient to mention an absolute improvement in spatial memory. In addition, there is no significant effect of COX inhibition by ibuprofen and nimesulide administration on NR2A and NR2B protein expressions. Prolonged administration period of these drugs may support improvement trend on spatial memory and may alternate NMDAR expressions. For this purpose, effects of chronic usage of these drugs on learning and memory should be investigated. 
